# Outcomes of Active Middle Ear Implants: Speech Perception and Quality of Life

**DOI:** 10.3390/jpm14080883

**Published:** 2024-08-21

**Authors:** Marzouqi Salamah, Athair Alradhi, Farid Alzhrani, Medhat Yousef

**Affiliations:** 1 King Abdullah Ear Specialist Center (KAESC), College of Medicine, King Saud University Medical City, King Saud University, Riyadh 11481, Saudi Arabia; faalzhrani@ksu.edu.sa (F.A.); medhatf78@yahoo.com (M.Y.); 2 MED-EL, GmbH, Riyadh 11563, Saudi Arabia; athair.alradhi@medel.com; 3 Audiology Unit, ENT Department, Menoufia University, Menoufia 32952, Egypt

**Keywords:** hearing loss, active middle ear implants, vibrant soundbridge, outcomes

## Abstract

Objective: To evaluate audiological outcomes, quality of life, and complications in patients implanted with Active middle ear implants (AMEI). The secondary objective is to investigate the required duration after implantation to reach satisfactory outcomes. Methods: This retrospective study included 31 patients implanted with Active middle ear implants (AMEI) with different methods of floating mass transducer attachment. Patients with incomplete medical records and those who did not respond to postoperative follow-up were excluded. Patients were assessed preoperatively, and at one, three, and six months postoperatively. The assessment included Pure Tone Average (PTA4), speech reception threshold (SRT), and speech discrimination score (SDS). The Speech Spatial and Qualities of Hearing scale (SSQ12) was also used to evaluate levels of satisfaction. Result: There are no significant differences found in PTA and SRT between the 3-, 6-, and 12-month visits. The speech reception threshold (SRT) showed a statistically significant improvement at 3, 6, and 12 months post-operative measures compared to pre-operative. Additionally, the SDS exhibited a significant increase only after 12 months, compared to the 3-month time point. However, satisfaction levels did not significantly differ between the 6-month and 12-month measurements following surgery. Conclusion: The Vibrant Soundbridge improves subjective satisfaction scores and audiological test scores in patients with different types of hearing loss. AMEI has a low risk of medical or surgical complications, the ease of using a hearing implant, and the social benefits of good hearing and communication.

## 1. Introduction

Numerous clinical studies have been carried out since the AMEI device was first introduced to show the advantages of the device for hearing-impaired patients [1,2]; AMEI use is known to benefit patients with all three categories of hearing loss, as established by Luetje et al. [3] for sensorineural hearing loss and Baumgartner et al. [4] for conductive and mixed hearing loss.

In addition to reducing quality of life, hearing loss has been linked to social isolation, depression, cognitive decline, and communication problems [5]. Consequently, there is a lot of interest in the rehabilitation of hearing loss in the elderly population [6].

Auditory rehabilitation (AR) is most frequently accomplished with hearing aids (HA). Some HA users have complained of aural feedback, occlusion effects, and pain from the device filling up their ears as a response [6]. As a result, alternatives have been created over the last two decades. In addition to cochlear implants, auditory brainstem implants, and bone conduction (BC) devices, the active middle ear implant (AMEI) is one of the most commonly utilized devices [7].

Growing technological advancements and surgical expertise in otological implantation have resulted in the field’s rapid growth and expansion. Particularly AMEIs have developed into a well-recognized rehabilitation technique to treat not only sensorineural hearing loss but also conductive hearing loss (CHL) or mixed hearing loss (MHL) in recent years by using different coupling options [7]. Ugo Fisch performed the first AMEI implantation in 1996, connecting the FMT to the incus. Since then, multiple coupling solutions have been developed for various middle ear problems [5,6].

Active middle ear implants (AMEIs) are a type of implantable hearing device that work by directly stimulating the structures of the middle and inner ear. They are designed to bypass the normal sound conduction pathway through the external and middle ear.

AMEIs achieve this by using either electromagnetic or piezoelectric mechanisms to induce vibrations in the ossicular chain or the oval/round window. The electromagnetic AMEIs, like the Esteem (Envoy Medical, White Bear Lake, MN, USA)™ and Carina (Cochlear, Sydney, Australia)™ systems are fully implantable with no external components. The VSB™ is the most commonly used AMEI and is a semi-implantable AMEI comprising both internal and external components [8].

The VSB™ is an implantable hearing implant that has been shown in numerous international tests to be effective and safe [9]. It is a good alternative to traditional hearing aids when they are unsatisfactory to patients, particularly when they do not provide enough hearing gain in noise or at high frequencies, or when they are contraindicated in patients for anatomical reasons or due to infection in the external ear canal [10]. VSB is an option for patients unable to undergo conventional surgery and those unsatisfied with their traditional hearing aids. The use of VSB in patients with radical cavities has demonstrated excellent results over the years [8].

The VSB consists of an external component, the audio processor (AP), and an implanted component, the vibrating ossicular prosthesis (VORP), which incorporates a receiver/stimulator, a conductor link, and a floating mass transducer (FMT).

The Vibrant Soundbridge (VSB) system offers various coupling options that allow the floating mass transducer (FMT) component to be attached at different locations, including the short process of the incus, long process of the incus, stapes, and round window. This provides a more customized approach to patients and expands the opportunities for using the VSB system in individuals with congenital aural atresia as well as those with acquired conductive or mixed hearing loss, such as following cholesteatoma surgery [8].

The AP sends information to the VORP, which causes the FMT to vibrate the mobile structure of the middle ear (i.e., incus, stapes superstructure, or the stapes footplate) or the inner ear (i.e., the round window membrane), stimulating the cochlear fluids [11].

Despite that AMEI has been used with patients for many years, there is still a lack of scientific literature within the area of the Middle East about the efficacy and performance of this device in implanted patients. Therefore, the objective of this study is to evaluate audiological outcomes, quality of life, and complications in patients implanted with AMEI. The secondary objective is to investigate the required duration after implantation to reach satisfactory outcomes.

## 2. Materials and Methods

This retrospective study included wholly patients who underwent active middle ear implantation with a Vibrant Soundbridge^TM^ device (VSB) (MED-EL, Innsbruck, Austria) at a single referral centre from August 2017 to October 2021. According to the manufacturer’s indication, the coupling modality was selected based on the patient’s middle ear anatomy and kind of hearing loss. This study was approved by the Institutional Review Board and done in accordance with the Declaration of Helsinki.

### 2.1. Participants

The study’s population was not limited by gender or laterality. The study included all patients who underwent middle ear implantation according to the criteria; patients with conductive (including those with aural atresia, microtia, and external canal stenosis) and mixed hearing loss, as well as those with moderate to severe sensorineural hearing loss that had been stable for at least two years and unaided speech discrimination scores (SDS) of 50% or better. Patients with incomplete medical records and those who did not respond to postoperative follow-ups were excluded. After treatment, all patients with hearing loss at our center received one hour-long session each week. Patients and their parents (in the case of children) participate in the sessions with the therapists.

### 2.2. Outcome Measures

All subjects underwent thorough audiological evaluations at two time points: (1) before surgery; (2) at the time of follow up at 3 months, 6 months, and 12 months postoperatively. The results were compared to detect changes in the hearing threshold.

Pure-tone audiometry was conducted, including ear-specific air conduction (AC) and bone conduction (BC). Pure-tone average (PTA4) values were calculated as the mean thresholds at 0.5, 1.0, 2.0, and 4.0 kHz. Aided speech audiometry was measured in the sound field for each patient through a loudspeaker placed at 45° azimuth. The hearing tests were measured with appropriate contralateral masking through calibrated headphones.

Speech testing was conducted and included the speech reception threshold (SRT) and speech discrimination scores (SDS) in quiet and in noise at 65 dB SPL. All speech tests were performed using spondee Arabic words and phonetically balanced Arabic words. The contralateral ear was masked during all hearing tests. The recommendations by the American Speech-Language and Hearing Association were used for all speech audiometry measurements [12].

### 2.3. Questionnaire

Noble et al. developed the SSQ12, which was a clinically useful short form of the original Speech, Spatial, and Qualities of Hearing Scale (SSQ). The goal of the SSQ12 was to compile a collection of relevant assessments that represented the full SSQ scale, providing clinicians and researchers with a practical and scaled-down version of the assessment tool [13].

The SSQ12, administered to the patients, is a hearing-specific questionnaire consisting of 12 questions about the ability to cope with different listening situations before and after an intervention [13]. The “benefit” version of this questionnaire (SSQ12) was specifically designed for retrospective assessment in cases in which no questionnaire was administered before intervention. The domains assessed by the SSQ12 include speech hearing (five questions), spatial hearing (three questions), and quality and ease of hearing (four questions). Each item of the SSQ12 can be rated on a 10-point scale ranging from −5 to +5. The midpoint (zero) corresponds to no change due to the intervention. The SSQ12 results in an overall score and scores in the three subscales (speech, spatial, quality).

## 3. Results

The study was carried out on 31 patients who used the AMEI system. About 54.8% of them were right-sided and the remaining 45.2% were left. Looking at the types of hearing loss, we found that 64.5% of patients had conductive, 22.6% had mixed, and 12.9% had sensorineural hearing loss. Furthermore, Clip Couplers were used in 41.9% of patients, SP Couplers in 38.7%, RWS Couplers in 16.1%, and the remaining 3.2% used LP Couplers. Regarding the duration of surgery, about 45.2% of patients spent 3 h, about 19.4% spent 2 and 2.5 h for both, about 12.9% spent 4 h, and the remaining 3.2% spent 5 h. The overall mean surgical duration was 2.9 ± 0.7 h.

The VORP template drawing was made, in 28.6%, before the incision. A total of 96.4% of patients passed the skin flap Gauge test (7 mm), while the remaining 3.6% were thinned. About 82.1% of patients had stapes bones and about 14.3% of them had limiting posterior tympanotomy, while the remaining 85.7% did not have any abnormal facial nerve course. About 76.5% of patients had a visible round window niche with a mean age at operation of 25.3 ± 18.9 years. Furthermore, only one patient showed postoperative secondary facial nerve paralysis.

The measured pre-operative air conduction pure-tone audiometry (PTA) was 65.5 ± 17.2 at 250 Hz; 64.0 ± 13.7 at 0.5 KHz; 59.4 ± 10.5 at 1 KHz; 56.0 ± 10.8 at 2 KHz; 66.0 ± 13.3 at 4 KHz; and 69.5 ± 15.5 at 6 KHz, while the mean PTA 4 was 61.3 ± 8.6. The pre-operative bone conduction pure-tone audiometry (PTA) was 13.7 ± 14.7 at 0.5 KHz; 13.9 ± 16.1 at 1 KHz; 24.2 ± 18.8 at 2 KHz; and 22.1 ± 21.9 at 4 KHz. The mean pre-operative Speech Reception Threshold was 59.4 ± 11.2, the mean pre-operative speech discrimination score was 36.8 ± 23.7% in quiet and 21.6 ± 15.0% in noise. The mean pre-operative Speech, Spatial, and Qualities of Hearing Scale was 4.9 ± 2.4 (Table 1).

### Outcome Measures

**Figure 1:**
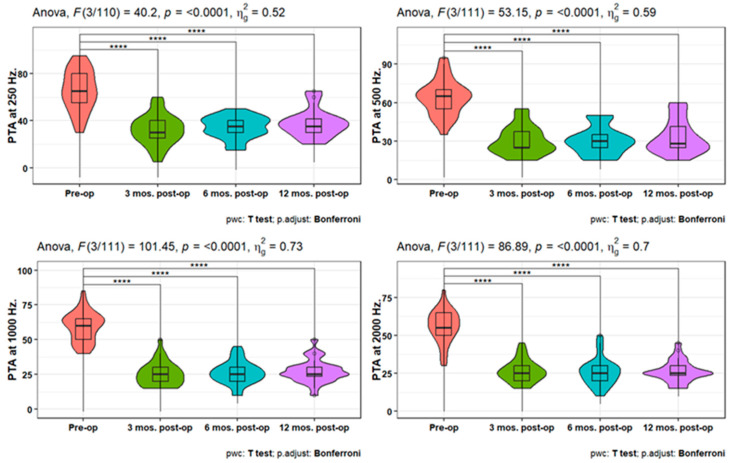
Improvement in PTA measures at 250, 500, 1000, and 2000 Hz. over 12 months post-operation. **** indicates statistical significance.

**Figure 2:**
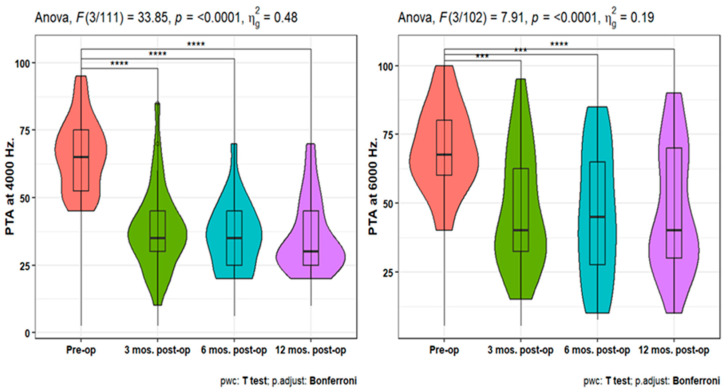
Improvement in PTA measures at 4000 and 6000 Hz. over 12 months post-operation. *** or **** indicates statistical significance.

**Figure 3:**
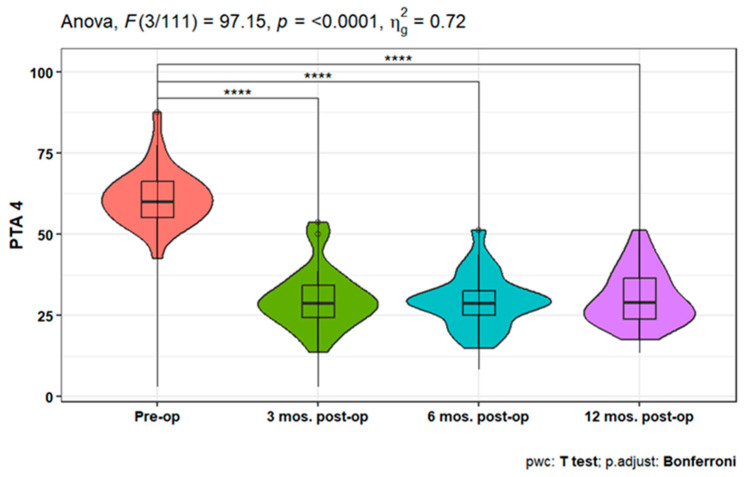
Improvement in PTA 4 over 12 months post-operation. **** indicates statistical significance.

**Figure 4:**
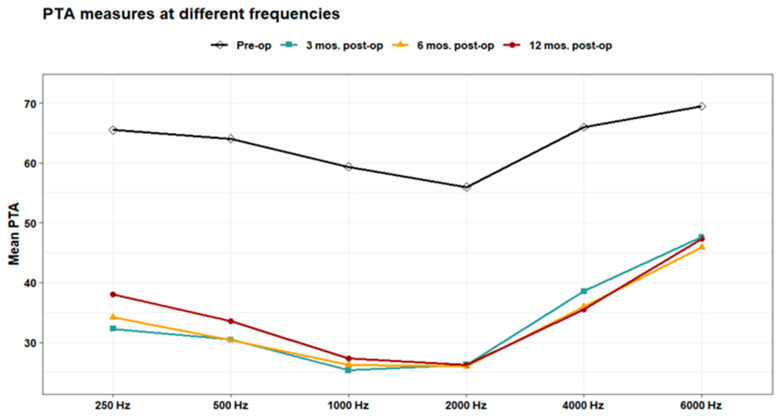
Mean PTA measures at different frequencies pre-operation and 3, 6, and 12 mos. post-operation.

Pure-tone audiometry (PTA) measures showed significant improvement (*p* < 0.001) at 3-, 6-, and 12-months post-operation compared to the pre-operative measures at 250, 500, 1000, 2000, 4000, and 6000 Hz. Also, PTA4 showed a significant improvement after 3-, 6-, and 12-months post-operation compared to the pre-operative one. However, the pairwise comparisons of post-operative PTA4 at the tested time points did not show significant differences (Figure 1, Figure 2 and Figure 3). The line chart in (Figure 4) also showed the non-significant differences between 3, 6, and 12 months post-operative PTA measures at different frequencies in contrast to the significant difference between PTA at every post-op time point compared to the pre-operative measures.

**Figure 5:**
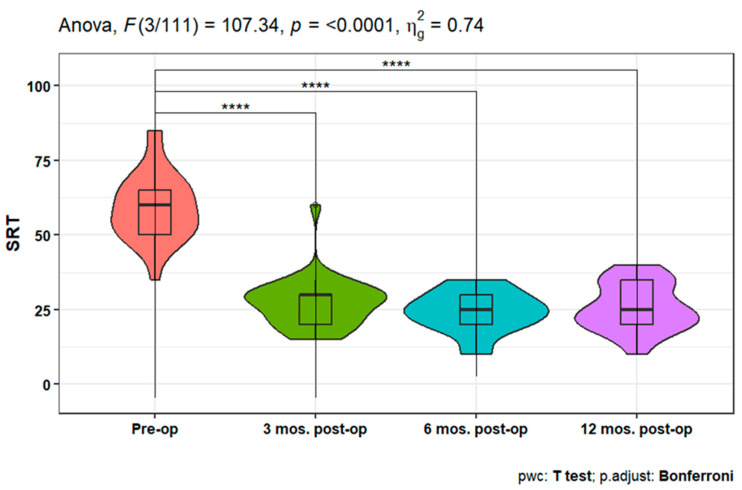
Improvement in speech reception threshold (SRT) Over 12 months post-operation. **** indicates statistical significance.

The speech reception threshold (SRT) showed a statistically significant improvement at 3-, 6-, and 12-months post-operative measures compared to pre-operative SRT (*p* < 0.001). Moreover, non-significant differences were detected between the three postoperative time points (Figure 5).

**Figure 6:**
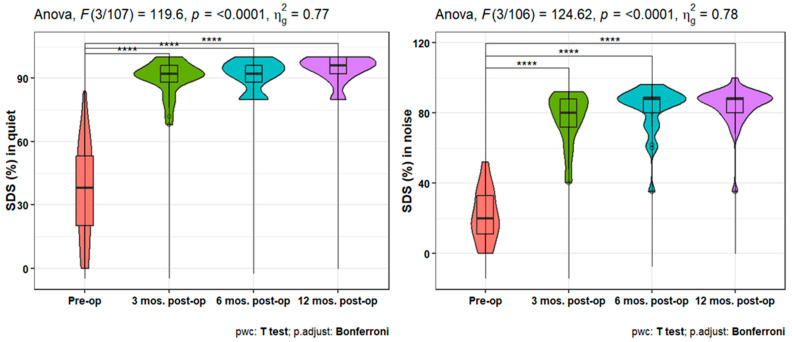
Improvement in speech discrimination scores (SDS) in quiet and noise over 12 months post-operation. **** indicates statistical significance.

**Figure 7:**
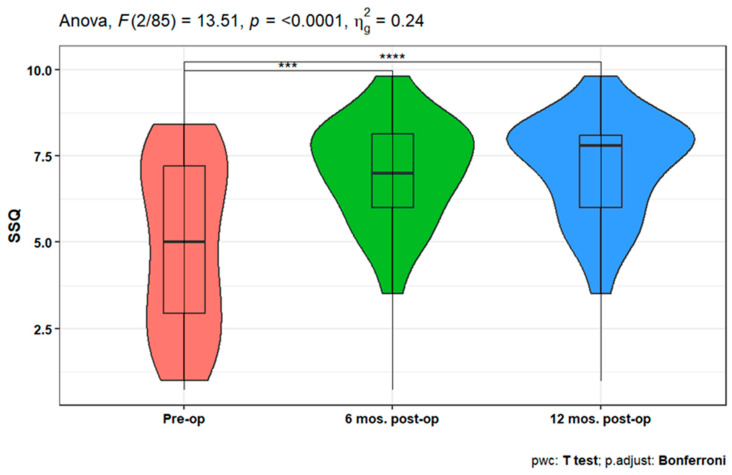
Improvement in Speech, Spatial and Qualities of Hearing Scale (SSQ) over 12 months post-operation. *** or **** indicates statistical significance.

Furthermore, the speech discrimination scores (SDS) showed a statistically significant increase at 3-, 6-, and 12-months post-operation either in quiet or in noisy conditions compared to the pre-operation ones (*p* < 0.001). In addition, there is a statistically significant increase in SDS in quiet conditions after 12 months compared to the 3 months post-operative scores (*p* = 0.021) (Figure 6). When studying the variation (in SSQ), the analysis showed a statistically significant increase after 6- and 12-months post-operation compared to the pre-operative scale (*p* < 0.001 for both). However, there is no significant difference between the SSQ at 6- and 12-months post-op (Figure 7).

## 4. Discussion

In multiple instances of auditory atresia and fibrous dysplasia of the temporal bone (FDTB), AMEI has been successfully used as one of the treatment options for individuals with conductive or mixed hearing loss, with outcomes that greatly improved PTA, SRT, and SDS [7,14].

According to preliminary evidence from existing patients, AMEI is a safe and effective therapeutic option for people with all three types of hearing loss etiologies. There were no major intraoperative or postoperative complications [7]. In this retrospective analysis, patients implanted with AMEI were evaluated for audiological outcomes, quality of life, and complications in a single-center study.

As analysis showed that patients’ perceptions of the AMEI benefits for PTA, SRT, and SDS were clearly improved, with most patients in this study reporting being generally satisfied or extremely satisfied with the device. For the group of 31 implanted patients, the audiological follow-up data revealed a significant improvement in hearing thresholds over time at 250 Hz, 500 Hz, 1000 Hz, 2000 Hz, 4000 Hz, and 6000 Hz. Additionally, PTA 4 showed a statistically significant decline post-operatively as compared to pre-operatively. However, there were no discernible variations across the postoperative time points in pairwise comparisons.

In terms of speech recognition outcomes, an audiologic benefit for AMEI revealed a statistically significant decrease in 3-, 6-, and 12-month post-operative measurements when compared to pre-operative SRT. Furthermore, despite the non-significant differences found between the three post-operative time points, Alzahrani et al. [15] report that SRT measurements with AMEI were significantly better in all patients compared to preoperative measurements. Ernst et al. [14] and Sprinzl et al. [7] reported speech recognition improvement of 52% to 81% after at least 6 months of use with Freiburger monosyllabic words, which is similar to what our patients demonstrated.

Lassaletta et al. [16] observed that after 1 year postoperation, word recognition (SDS at 65 dB) greatly improved; also, Alzahrani et al. [15] report the mean SDS in quiet conditions were 51% in the unaided condition and 94.60% in the aided condition for monosyllables at 65 dB HL. Our results indicated a significant increase in post-operative speech in either quiet or noise at 3, 6, and 12 months, compared to pre-operative. In addition to a statistically significant rise in SDS in quiet conditions after 12 months, compared to 3 months post-operative scores (*p* = 0.021). As a result, we infer that the AMEI improves speech understanding outcomes significantly.

In this study, assessing patient benefit following AMEI implantation with the SSQ, a significant increase after 6- and 12-months post-operation was revealed, compared to the pre-operative scale; but, there was no significant difference between the 6- and 12-month post-operative SSQ. This supports the concept that patients who improve their speech understanding with AMEI also have higher hearing quality, at least in the categories addressed by the SSQ.

According to these results, the patient’s subjective reports of an improvement in their hearing and overall quality of life may be supported by actual improvement. The long-term postoperative outcomes of the AMEI users were published by Rameh et al. [17] in their study. Although there was a lot of variety among the devices, patients were generally happy with their implants. Positive effects on patients’ social interactions are seen when they can hear and speak clearly. Due to communication challenges, people who have hearing loss frequently withdraw from social situations, particularly ones with background noise.

In addition to the audiologic findings, we discovered no differences in perioperative and postoperative complications, which is consistent with the literature [1,18,19]. However, only one patient experienced postoperative secondary facial nerve paralysis, despite there being no history of heating, or manipulation of the facial nerve during the surgery. We hypothesized that this complication could have been caused by a late reactivated viral infection, such as from herpes simplex virus 1 or varicella zoster virus, and it resolved spontaneously without any further intervention.

In our investigation, all patients exhibited good, aided hearing thresholds with the AMEI following surgery, demonstrating that the surgical and fitting methods were adequate for the patient’s demands, as in Wolf-Magele et al. [20].

An average postoperative complication rate of 16.3% was found in a systematic review of the use of the AMEI for treating conductive and mixed hearing loss [14]. The reported explanation percentage for AMEI, however, in long-term follow-up studies of AMEI use in individuals with mixed hearing loss, ranged from 10.17% to 18.5% [21,22]. Colletti et al. [23] described two explanations that were required due to misdiagnosed significant hearing loss. Brkic et al. [21] reported a 10.2% explanation rate.

Revision surgery was reported in two of our patients, one of who had a history of CWD and was presented with electrode protrusion into the external auditory meatus 2 years after the primary surgery. The second patient presented with decreased performance one year post implantation. During the revision surgery, the FMT was found to have limited movement due to mastoid bone re-growth around it. Device failure was reported by Zwartenkot et al. [22] (7% technical failure rate), Brkic et al. [21] (4.0%), Sprinzl et al. [7], and Schmuziger et al. [24]; however, none of our patients experienced device failure.

The main limitation of the study was that all questionnaires were administered only after treatment (i.e., after AMEI). The second limitation was the inability to compare the AMEI outcome with conventional hearing aids. Despite possible limitations, retrospective studies generally reflect clinical practice in terms of patient selection, assessment, and surgical techniques and are therefore generalizable to routine clinical care.

After analyzing the temporal improvement in outcome measures, we found that no significant differences were found in PTA and SRT between the 3-, 6-, and 12-month visits. Consequently, it is reasonable to suggest that these outcomes could be assessed once or twice per year. Additionally, the SDS exhibited a significant increase only after 12 months, compared to the 3-month time point, which further supports the notion that measuring the SDS at 6 months may be of lesser importance. However, satisfaction levels did not significantly differ between the 6-month and 12-month measurements following surgery. As a result, these findings warrant further investigation to establish the necessity of a 6-month visit after AMEI, or if it suffices to have patients visit the clinic at 3- and 12-month intervals post-surgery. That helps patients with time, especially patients who stay far away from the hospital.

## 5. Conclusions

This retrospective study showed that AMEI can improve subjective satisfaction scores and audiological test scores in patients with different types of hearing loss. As AMEI has a low risk of medical or surgical complications, the ease of using a hearing implant, and the social benefits of good hearing and communication, we think that AMEI should be regularly offered to patients with hearing loss if it is audiological and surgically indicated.

## Figures and Tables

**Table 1:** Baseline patients’ demographics, surgical characteristics, and non-aided pre-operative pure-tone audiometry measures.

Demographics and Surgical Characteristics		Overall N = 31
Side	Left	14 (45.2)
	Right	17 (54.8)
Type of HL	CHL	20 (64.5)
	MHL	7 (22.6)
	SNHL	4 (12.9)
Coupler	Clip Coupler	13 (41.9)
	LP Coupler	1 (3.2)
	RWS Coupler	5 (16.1)
	SP Coupler	12 (38.7)
Surgical Time (Hours)	2:00:00	6 (19.4)
	2:30:00	6 (19.4)
	3:00:00	14 (45.2)
	4:00:00	4 (12.9)
	5:00:00	1 (3.2)
	Mean (SD)	2.9 (0.7)
VORP template drawing made before incision	NO	20 (71.4)
	YES	8 (28.6)
Skin flap measured with skin flap gauge 7	Pass	27 (96.4)
	Thinned	1 (3.6)
Stapes present	NO	5 (17.9)
	YES	23 (82.1)
Abnormal facial nerve course	NO	24 (85.7)
	Limiting Posterior Tympanotomy	4 (14.3)
RW niche visible	NO	4 (23.5)
	YES	13 (76.5)
Age at operation (Years)	Mean (SD)	25.3 (18.9)
Postoperative complications	None	23 (95.8)
	Secondary facial nerve paralysis	1 (4.2)
Pure-Tone Audiometry (PTA) Air Conduction		
250 Hz	Mean (SD)	65.5 (17.2)
500 Hz	Mean (SD)	64.0 (13.7)
1000 Hz	Mean (SD)	59.4 (10.5)
2000 Hz	Mean (SD)	56.0 (10.8)
4000 Hz	Mean (SD)	66.0 (13.3)
6000 Hz	Mean (SD)	69.5 (15.5)
PTA4	Mean (SD)	61.3 (8.6)
Bone Conduction		
500 Hz	Mean (SD)	13.7 (14.7)
1000 Hz	Mean (SD)	13.9 (16.1)
2000 Hz	Mean (SD)	24.2 (18.8)
4000 Hz	Mean (SD)	22.1 (21.9)
Speech Audiometry Measures		
SRT	Mean (SD)	59.4 (11.2)
SDS in Quite (score %)	Mean (SD)	36.8 (23.7)
SDS in Noise (score %)	Mean (SD)	21.6 (15.0)
SSQ	Mean (SD)	4.9 (2.4)

Data are represented as count (%), mean (standard deviation).

## Data Availability

The data that support the findings of this study are available from the corresponding author, upon reasonable request.
